# Introduction of gastric endoscopic submucosal dissection and skill acquisition in a regional hospital

**DOI:** 10.1002/jgh3.12249

**Published:** 2019-08-27

**Authors:** Junichi Fujiwara, Satohiro Matsumoto, Kenichi Yamanaka, Masanari Sekine, Takehiro Ishii, Takuma Ajimine, Hirosato Mashima

**Affiliations:** ^1^ Department of Gastroenterology, Saitama Medical Center Jichi Medical University Saitama Japan; ^2^ Department of Gastroenterology Kitaakita City Hospital Akita Japan

**Keywords:** endoscopic submucosal dissection, learning curve, regional hospital

## Abstract

**Background and Aim:**

Endoscopic submucosal dissection (ESD) is standard treatment for early gastric cancer. With aging of the population in Japan being more pronounced in rural areas, the availability of ESD at regional hospitals is becoming important. Here, we assessed the learning curve of one physician for skill acquisition in gastric ESD.

**Methods:**

The subjects were 34 patients (38 lesions) who underwent gastric ESD at a regional hospital between October 2014 and March 2017 and 15 patients (15 lesions) who underwent the procedure at a university hospital between April 2017 and April 2018. The resection periods of the first 19 lesions and subsequent 19 lesions at the regional hospital were defined as the first and seconds periods, and the resection period of 15 lesions at the university hospital was defined as the third period. The learning curve across the three periods was assessed using the cumulative sum analysis method.

**Results:**

The resection speed in the first, second, and third periods were 6.4 ± 4.1, 6.9 ± 3.4, and 9.4 ± 5.4 mm^2^/min, respectively (not significant). The slope of the learning curve began to increase at the 30th patient. The en bloc resection and curative resection rates did not differ significantly among the three periods. There were no serious procedure‐related complications.

**Conclusion:**

This study showed that the introduction of gastric ESD at a regional hospital is possible, and that a certain skill level was acquired by the 30th patient. Furthermore, with careful patient selection, favorable results can be obtained and procedural safety ensured.

## Graphical abstract

The introduction of gastric endoscopic submucosal dissection at a regional hospital is possible. Furthermore, with careful patient selection, favorable results can be obtained and procedural safety ensured.

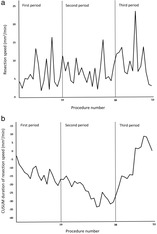

## Introduction

Endoscopic submucosal dissection (ESD) was developed as an effective treatment method for early gastric cancer.^1^, ^2^ Many studies have since reported the effectiveness of ESD, and ESD has been established as a standard treatment for early gastric cancer.^3^, ^4^, ^5^ However, ESD is a technically challenging procedure. It takes a considerable time for operators to acquire the skill for ESD, and there is a risk of complications. Therefore, specialized centers would be considered the ideal places to acquire the skill.^6^ Japan is facing the problem of an aging population. According to a nationwide survey conducted in 2014, approximately 80% of gastric cancer patients were aged 65 years or older, and 14–18.7% of patients undergoing gastric ESD were aged 80 years or older.^7^, ^8^ However, elderly patients often have difficulty in visiting highly specialized centers due to the lack of suitable transportation or other reasons, such as the person(s) accompanying him/her living at a distant location. On the other hand, facilities for ESD are not available in many regions, especially in remote areas and islands, where shortage of physicians is common. Therefore, if facilities for ESD were available and acquisition of skill for the procedure was possible at regional hospitals, it would prove beneficial for both patients and physicians. In particular, it may contribute to improvement of treatment for early gastric cancer in the setting of local health care and decrease the mortality due to gastric cancer.

Many studies have reported on the learning curve for the acquisition of the skill in performing ESD, but few studies have reported on the learning curve for ESD skill acquisition at regional hospitals with newly introduced facilities for ESD.^9^, ^10^, ^11^In this study, we assessed the learning curve for acquiring the skill in performing gastric ESD and the treatment results of a single endoscopist who worked at a regional hospital and then at a university hospital and evaluated the feasibility and safety of introducing gastric ESD in regional hospitals.

## Methods

### *Subjects*

The subjects were 34 patients (38 lesions) who underwent ESD for gastric tumor(s) at a regional hospital (Kitaakita City Hospital) between October 2014 and March 2017 and 15 patients (15 lesions) who underwent the procedure at a university hospital (Saitama Medical Center, Jichi Medical University) between April 2017 and April 2018. Most of the ESD procedures (96%) were performed by a single endoscopist. In the assessment of the learning curve for ESD, the periods during which the first 19 procedures and the remaining 19 procedures were performed at the regional hospital were defined as the first and second periods, respectively, and the period during which the 15 procedures were performed at the university hospital was defined as the third period. Procedures 3 and 4, procedures 16 and 30, procedures 27 and 31, and procedures 32 and 33 were conducted on the same patients. Procedures 3 and 4 and procedures 32 and 33 were performed on the same day. The background characteristics of the lesion, resection speed, diameter of the resected specimen, en bloc resection rate, curative resection rate, and rate of complications were compared among the three periods. Patients in whom the ESD was aborted (procedure 5) and in whom the operator was changed (procedures 40 and 50) were excluded from the analysis of the resection speed. The resection speed was calculated using the following formula: largest diameter of the resected specimen (mm) × shortest diameter of the resected specimen (mm) × 3.14 × 0.25/resection time (min).

### *Operators*

At the time of this study, the physician who performed the ESDs had 5 years' experience as an endoscopist. The endoscopist had received training on ESD at Akita University Hospital between October 2013 and March 2014. During the training program, he had received training on the techniques for ESD and participated in ESD study groups, as appropriate. After receiving the training, he had performed the procedure in 20 cases as an expert assistant. During the latter period, he also experienced actually performing the ESD procedure as the operator, under supervision, in 10 cases. However, in these cases, after 30–60 min of the procedure, an expert took over the procedure.

### *Lesion characteristics and curability*

All lesions were histopathologically classified according to the Japanese Classification of Gastric Carcinoma.^12^ The macroscopic type was classified as the protruded type, flat type, or depressed type. The size of the tumor and the resected area were measured from the resected specimen. The tumor location was classified as the upper third, middle third, or lower third of the stomach. The depth of tumor invasion was classified as pT1a (up to the mucosa) or pT1b (up to the submucosa). Invasion of the submucosal layer (SM) was divided into SM1 (less than 0.5 mm from the muscularis mucosae) and SM2 (submucosal invasion depth more than 0.5 mm). The tumor differentiation grade was based on the most dominant differentiation grade, and the tumors were classified as adenoma, differentiated cancer (including well‐differentiated, moderately differentiated, tubular, or papillary adenocarcinoma), or undifferentiated cancer (poorly differentiated adenocarcinoma or signet ring cell carcinoma).

En bloc resection was defined as resection in a single piece. Complete resection was defined as en bloc resection of a tumor with a negative horizontal margin and vertical margin. Curative resection was defined as follows: en bloc resection, tumor size ≤2 cm, differentiated‐type tumor, pT1a, ulceration (Ul)‐negative, no lymphovascular invasion (ly(−), v(−)), negative horizontal margin (HM0), and negative vertical margin (VM0). The expanded indications of curative resection were as follows: en bloc resection, ly(−), v(−), HM0, and VM0, as well as (a) tumor size ≥2 cm, differentiated‐type tumor, pT1a, Ul(−); (b) a tumor size of ≤3 cm, differentiated‐type tumor, pT1a, Ul(+); (c) a tumor size of ≤2 cm, undifferentiated‐type tumor, pT1a, Ul(−); and (d) a tumor size of ≤3 cm, differentiated‐type tumor, pT1b (SM1).^13^, ^14^ All other lesions were classified as noncurative resection.

### *Adverse events*

Postoperative bleeding was defined as bleeding events necessitating endoscopic hemostasis, including hematemesis and/or melena, after the procedure or a decrease of the hemoglobin level by more than 2 mg/dL compared to the preoperative hemoglobin level.

### *ESD procedure*

At Kitaakita City Hospital, ESD was performed with a conventional single‐channel endoscope (GIF‐HQ290; Olympus, Tokyo, Japan) and the high‐frequency generator ESG‐100 (Olympus). Nurses assisted the endoscopist in each procedure. Midazolam was used for sedation. The gastric mucosal incision was made mainly using the IT2‐Knife (KD‐611L; Olympus) and a needle‐shaped scalpel. Peeling of the gastric submucosal layer was performed using the IT2‐Knife or Dual‐knife (KD‐650L; Olympus). The injection solution was prepared by mixing normal saline with 0.4% sodium hyaluronate solution (Mucoup; Johnson and Johnson, Tokyo, Japan) at the ratio of 3:7 and adding indigo carmine. Hemostasis was performed using high frequency‐type coagulation forceps, the Coagrasper (FD‐411QR; Olympus). The same endoscopist performed all the procedures. If the endoscopist could not complete the procedure, the treatment was discontinued, and then, the patient was considered for elective surgery. A second‐look upper endoscopy was performed on the day after the operation. If bleeding or an exposed vessel was observed, hemostasis was performed.

At Jichi Medical University Saitama Medical Center, ESD was performed with a conventional single‐channel endoscope (GIF‐Q260; Olympus) or a multibending scope (GIF‐2TQ260M; Olympus), depending on the lesion to be resected, using the high‐frequency generator VIO 300D (Erbe, Tubingen, Germany). Another physician assisted the endoscopist for each procedure. Flunitrazepam and pethidine hydrochloride were used for sedation. If sedation was insufficient, haloperidol was used as needed. The injection solution was prepared by mixing 10% glycerin and 0.4% sodium hyaluronate solution at the ratio of 1:1 and adding indigo carmine plus 0.01% epinephrine. Gastric mucosal incision and peeling of the submucosal layer were performed using a Dual knife or Dual knife J. Hemostasis was performed using Coagrasper. The operator could be changed at the discretion of a senior physician. A second‐look upper endoscopy was performed on the day after the operation. If bleeding or an exposed vessel was observed, hemostasis was performed.

### *CUSUM analysis*

In recent years, cumulative sum (CUSUM) analysis has been used for the quantitative assessment of learning curves for various procedures.^15^, ^16^ This method was introduced in the medical field in the 1970s for analysis of the learning curves for surgical procedures.^17^, ^18^ CUSUM is applied to show the sequential differences between each data point and the mean of all data points. For example, if the CUSUM value for procedure 1 is represented by C1, the resection speed in procedure 1 is represented by S1, and the mean resection speed of all procedures is denoted by Sm, then the CUSUM value is calculated by the following formula: C1 = S1 − Sm; the CUSUM value for procedure 2, represented by C2, could be calculated by adding C1 to the difference between S2 (resection speed in procedure 2) and Sm, as follows: C2 = C1 + (S2 − Sm) = S1 + S2‐2xSm. Accordingly, the CUSUM value of procedure *n* would be calculated by the formula: Cn = S1 + S2 + … + Sn − n_1_xSm. In this study, the ESD was discontinued in the fifth procedure, and the endoscopist was changed in the 40th and 50th procedures. Therefore, these three procedures were excluded from the analysis of the resection speed. Accordingly, the values for procedures 1–4 were calculated as *n*_1_ = *n*, those for procedures 6–39 as *n*_1_ = *n* − 1, those for procedures 41–49 as *n*_1_ = *n* − 2, and those for procedures 51–53 as *n*_1_ = *n* − 3. C_53_ = 0.

### *Statistical analysis*

A comparison of continuous variables among the three periods was performed using the Kruskal‐Wallis test. Comparison of ratios among the three periods was performed using Fisher's exact test. The significance level was set at *P* < 0.05. Statistical analyses were performed using the EZR software. The software is for R. More precisely, it is a modified version of R commander designed to add statistical functions frequently used in biostatistics.^19^

## Results

### *Patient and tumor characteristics*

There were no significant differences in the mean age or gender ratio of the subjects among the three periods. Complications and use of antithrombotic drugs tended to be observed more frequently in the second period, but the difference among the three periods was not significant (Table 1). The largest tumor diameter was 12.6 ± 5.6 mm in the first period, 18.4 ± 7.8 mm in the second period, and 14.5 ± 5.7 mm in the third period, with a significant difference among the three periods (*P* = 0.02). The percentage of cases with the tumor location in the U region was 5.3% in the first period, 5.3% in the second period, and 33.3% in the third period, with a significant difference among the three periods (*P* = 0.02). With regard to the macroscopic classification, type 0‐IIa lesions were more frequent in the first and second periods, while type 0‐IIc lesions were significantly more frequent in the third period. There were no significant differences in the distribution of the histological type or invasion depth among the three periods (Table 2).

**Table 1:** Interphase comparisons of patient characteristics

	First period (*n* = 17)	Second period (*n* = 18)	Third period (*n* = 15)	*P* value
Age (years)	76 ± 9	77 ± 8	75 ± 4	NS
Gender				
Male	9	11	10	NS
Female	8	7	5	NS
Baseline disease				
Hypertension	4	3	3	NS
Diabetes	3	4	1	NS
Cardiovascular disease	3	9	1	NS
Cerebrovascular disease	2	3	1	NS
Renal dysfunction	1	1	0	NS
Antithrombotic drugs	6	7	2	NS

NS, not significant.

**Table 2:** Interphase comparisons of tumor characteristics

	First period (*n* = 19)	Second period (*n* = 19)	Third period (*n* = 15)	*P* value
Tumor long axis (mm)	12.6 ± 5.6	18.4 ± 7.8	14.5 ± 5.7	0.02
Location				
Upper third	1	1	5	0.03
Middle third	3	7	2	NS
Lower third	15	11	8	NS
Macroscopic type				
0‐IIa	13	10	4	NS
0‐IIb	1	0	0	NS
0‐IIc	4	5	9	0.04
0‐IIb + IIa	1	1	0	NS
0‐IIa + IIc	0	1	2	NS
0‐I	0	2	0	NS
Histological type				
Adenoma	1	2	0	NS
Differentiated type	16	17	15	NS
Undifferentiated type	1	0	0	NS
Invasion depth				
Mucosa	17	18	14	NS
Submucosa	1	1	1	NS

NS, not significant.

### *Results of the CUSUM analysis*

**Figure 1:**
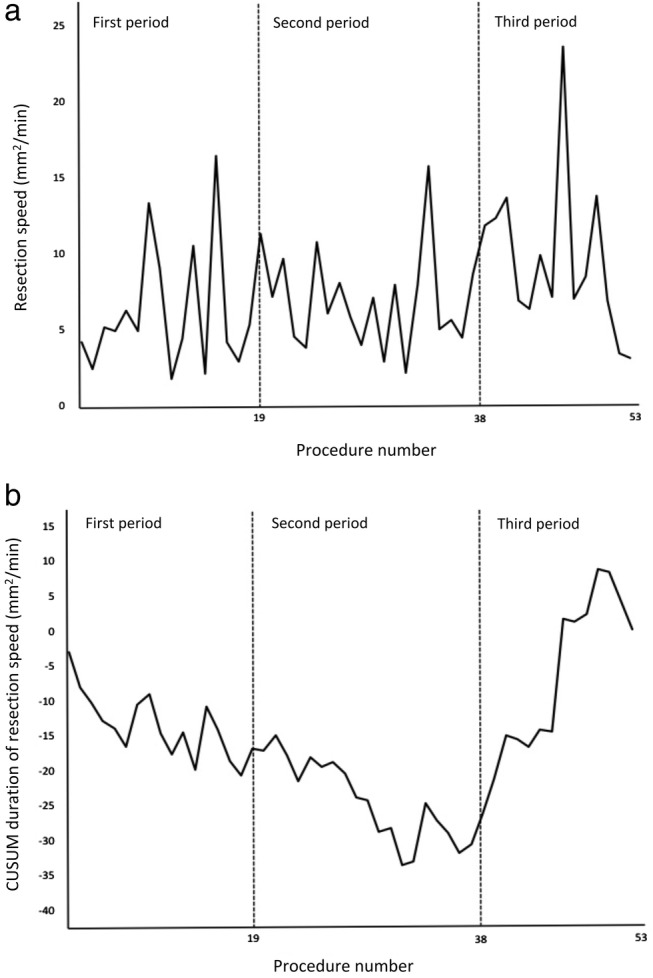
(a) Resection speed (mm^2^/min) against procedure number. (b) Cumulative sum (CUSUM) resection speed (mm^2^/min) against procedure number. First period was the first 19 procedures and second period was the second 19 procedures performed at regional hospital. Third period was the last 15 procedures performed at university hospital. Incomplete resection case (procedure 5) and operator‐changed cases (procedures 40 and 50) were excluded.

**Figure 2:**
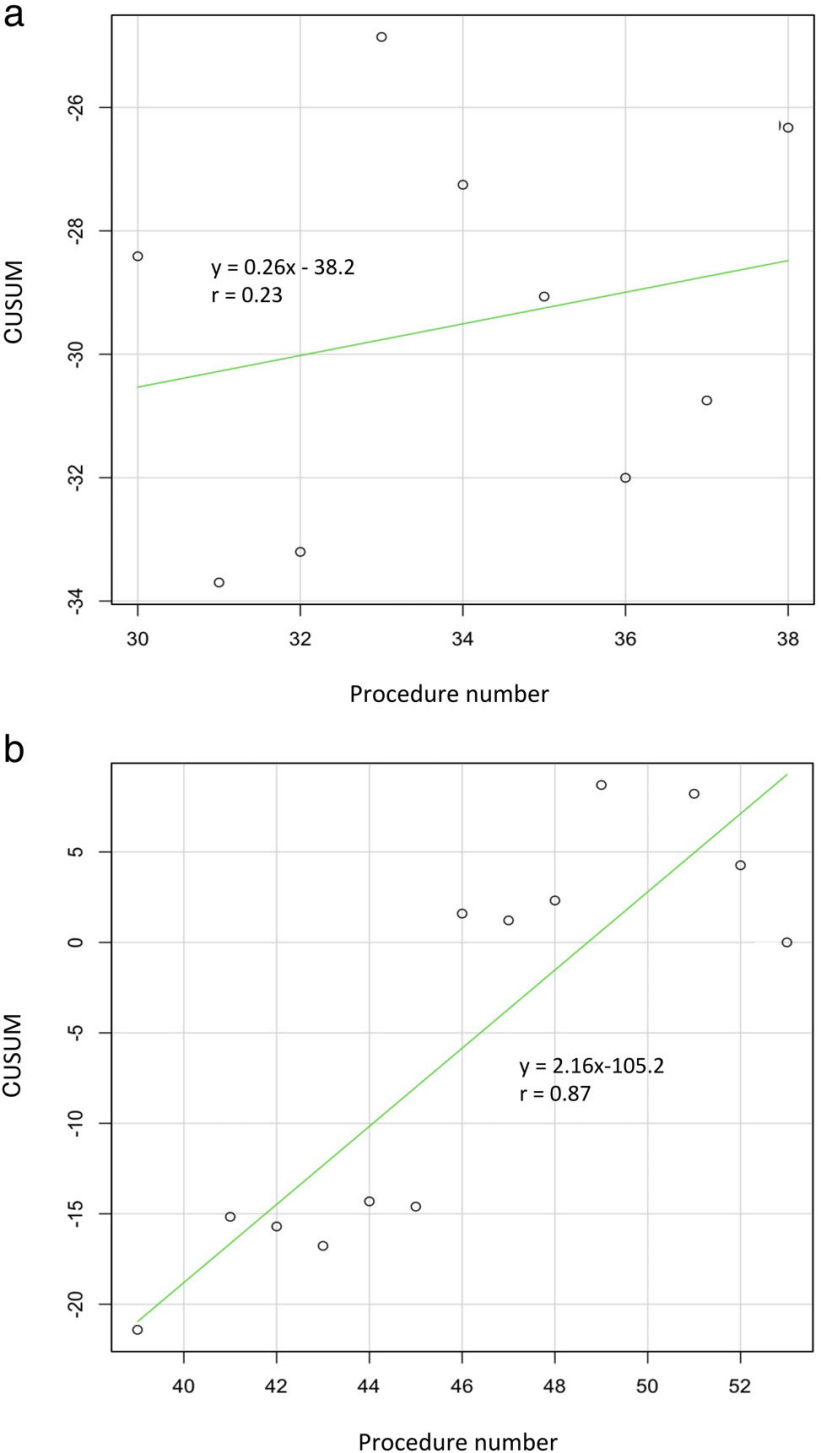
Lines of best fit for the last 23 procedures. A shows the procedure number of 30–38 performed at regional hospital. B shows the procedure number of 39–53 performed at university hospital. Incomplete resection case (procedure 5) and operator‐changed cases (procedures 40 and 50) were excluded. CUSUM, cumulative sum.

Figure 1 shows the resection speed and the learning curve. The resection speed varied among patients. The slope of the learning curve began to increase from the 30th procedure. The learning curve was steeper during the period at the specialized center (third period). The slopes of the regression curves by the CUSUM of procedures 30–38 and procedures 38–53 were 0.26 (r = 0.23) and 2.16 (r = 0.87), respectively (Fig. 2).

### *Clinical outcomes and complications*

Table 3 shows the treatment results and complications during the first, second, and third periods. There were no significant differences in the en bloc resection rate or curative resection rate among the three periods. The resected specimen area and resection time increased significantly across the three periods. The resection speed during the first, second, and third periods were 6.4 ± 4.1, 6.9 ± 3.4, and 9.4 ± 5.4 mm^2^/min, respectively, showing a tendency toward increase, although the difference was not significant. ESD procedures for lesions located in the upper third of the stomach take a longer time. The presence of a relatively greater number of lesions in the upper third during the third period could have introduced bias in this study. Therefore, we performed subgroup analysis for lesions at each location of the stomach (Table 4). The resection speed and specimen area increased significantly across the three periods for lesions in the lower third of the stomach. However, there were no significant differences in these parameters between lesions in the upper and middle third of the stomach. ESD was discontinued in the fifth procedure during the first period. The endoscopist was changed for two procedures during the third period. Perforation was observed during the ninth procedure in the first period, and postoperative bleeding was observed during procedures 21 and 38 in the second period; however, none of the patients showed any serious complications. The perforation that occurred during the ninth procedure in the first period healed with conservative treatment.

**Table 3:** Interphase comparisons of ESD procedural data

	First period (*n* = 19)	Second period (*n* = 19)	Third period (*n* = 15)	*P* value
En block resection rate (%)	95†	100	100	NS
Curative resection rate (%)	94†	95	93	NS
Specimen area (mm^2^)	262 ± 161†	506 ± 265	797 ± 395	<0.01
Resection time (min)	48 ± 29	87 ± 50	116 ± 83	<0.01
Resection speed (mm^2^/min)	6.4 ± 4.1†	6.9 ± 3.4	9.4 ± 5.4‡	NS
Complications				
Perforation	1	0	0	—
Bleeding	0	2	0	—
Interrupted operation	1	0	0	—
Operator change	0	0	2	—

† Excluding incomplete resection case (procedure 5).

‡ Excluding operator‐changed case (procedures 40 and 50).

—, not analyzed because the number is small; ESD, endoscopic submucosal dissection; NS, not significant.

**Table 4:** Comparison of each location of tumors according to the periods

	First period	Second period	Third period	*P* value
Upper third (*n*)	1	1	4†	
Specimen area (mm^2^)	314	471	624	NS
Resection time (min)	35	60	106	NS
Resection speed (mm^2^/min)	9.0	7.9	4.8	NS
Middle third (*n*)	2‡	7	2	
Specimen area (mm^2^)	247	707	526	NS
Resection time (min)	43	95	75	NS
Resection speed (mm^2^/min)	6.0	6.0	7.0	NS
Lower third (*n*)	15	11	7†	
Specimen area (mm^2^)	236	361	824	0.01
Resection time (min)	40	70	60	NS
Resection speed (mm^2^/min)	4.8	5.7	12.3	<0.01

† Excluding operator‐changed case (procedures 40 and 50).

‡ Excluding incomplete resection case (procedure 5).

NS, not significant.

## Discussion

When introducing ESD in regional hospitals, it is of utmost importance to ensure that there are (i) sufficient discussions with the medical staff, (ii) close collaboration with the department of surgery, and (iii) careful selection of patients for ESD. At Kitaakita City Hospital, conventional gastrointestinal endoscopic examination was already available when ESD was introduced in October 2014. Therefore, sufficient discussion was possible before the introduction of ESD. The discussion included the procedural aspects, staffing logistics, treatment hours, preparation of instruments (scope, high‐frequency device, knife, hemostasis device, etc.), and troubleshooting, and consensus was obtained from the staff. As the ESD procedure was performed by a physician, support from the department of surgery was essential. Close communication was maintained with the surgeons, and the schedules for ESD were adjusted in order to be prepared for any emergencies. Indications for ESD were determined according to the Japanese Classification of Gastric Carcinoma^12^; however, in principle, patients in whom the procedure was technically difficult and those with a high risk of complications were referred to specialized centers. ESD at the regional hospital was scheduled for patients in whom the procedure was not expected to be challenging, those who were considered low‐risk cases, and those who strongly desired to undergo treatment at the regional hospital. The procedure was performed after obtaining informed consent.

This study suggests that the basic skill for ESD can be acquired even in regional hospitals. CUSUM analysis showed that the average number of cases needed to acquire sufficient skill was 30. Yoshida *et al*. and Yamamoto *et al*. also reported that the average number of cases required for acquiring sufficient skill in gastric ESD was approximately 30.^10^, ^11^ In addition, Choi *et al*. reported that the number of cases required for acquiring sufficient skill for the safe performance of endoscopic mucosal resection was 40.^9^ Based on these findings, it is considered reasonable to assume that the number of procedures required for acquiring a sufficient level of skill for ESD is 30 and that the acquisition of a certain skill for ESD can be predicted from the experience of performing the procedure in a certain number of cases.

Jeon *et al*. and Tsou *et al*. reported that the resection speed in ESD differed significantly depending on the duration of learning.^20^, ^21^ In this study, the resection speed was not significantly different among the three periods, but it tended to increase with experience, which was supported by the results of an analysis of the learning curve. The slope decreased during the first period; plateaued from the 30th patient; and then started to increase, continuing into the third period. As shown in Figure 2, the slope after the 39th patient at the specialized center was steeper than the slope for the 30–38th procedure at the regional hospital. Ahn *et al*. reported that the tumor location may be a useful predictor of the time required to perform ESD.^22^ In this study, the resection speed increased significantly across the three periods, although the resection time was not significantly different for lesions in the lower third of the stomach. This suggested that experience at the high‐volume center contributed more to improvement of the resection speed, although there is also the possibility that the skill improvement at the regional hospital played a role in the increase of the resection speed in the later period.

Tsuji *et al*. reported that the learning of ESD under the supervision of experts in specialized centers is advantageous in that it prevents poor outcomes.^23^ However, our study suggests that the skill for ESD can be acquired while obtaining favorable outcomes even at a regional hospital, which is noteworthy.

Ojima *et al*. reported a high perforation rate in patients with lesions in the greater curvature, scar lesions, long resection times, and gastric remnant and a high postoperative bleeding rate in patients on antithrombotic agents, dialysis, and antihypertensive drugs.^24^ Nagata *et al*. reported that the factors with a negative impact on ESD performance were lesions in the upper third of the gastric body, presence of fibrosis, and large size of the resected tumor.^25^ In addition, Ono *et al*. argued that it would be more reasonable for beginners of gastric ESD to treat lesions in the lower part of the gastric body first and then successively treat those in the middle and upper third of the gastric body.^26^ Based on these findings, in the practice of ESD at regional hospitals, it would be desirable to select patients who are not taking anticoagulant drugs, have few complications, have relatively small lesions, have lesions in the lower part of the gastric body, and have lesions without fibrosis. For this study, patients meeting the above criteria were primarily selected in order to avoid challenging cases because a single endoscopist performed the procedure at the regional hospital. However, it was difficult to exclude all patients who met the exclusion criteria because many of the patients at the regional hospital were old and had complications and/or were taking antithrombotic drugs. With regard to the treatment results, the curative resection rate was 94% in the first period, 95% in the second period, and 93% in the third period, and the overall complication rate was 5.7%; these results were not significantly different from those published until now and are considered to be favorable.^27^

The limitations of this study included its retrospective design, the small sample size, differences in the target lesions and operation environment, and evaluation of a single endoscopist.

In conclusion, acquisition of the skill for gastric ESD is possible even at regional hospitals. In particular, cooperation with the medical staff and surgeons is important. Although training for the ESD procedure at a specialized center may be favorable, careful patient selection for ESD is still needed to ensure safety, acquisition of skills, and good treatment outcomes even at regional hospitals.
